# Non-operative management of posterior tibialis tendon dysfunction: design of a randomized clinical trial [NCT00279630]

**DOI:** 10.1186/1471-2474-7-49

**Published:** 2006-06-06

**Authors:** Kornelia Kulig, Amy B Pomrantz, Judith M Burnfield, Stephen F Reischl, Susan Mais-Requejo, David B Thordarson, Ronald W Smith

**Affiliations:** 1 Associate Professor of Clinical Physical Therapy, Department of Biokinesiology and Physical Therapy, University of Southern California, Los Angeles, CA, USA; 2 Orthopedic Physical Therapy Resident, Department of Biokinesiology and Physical Therapy, University of Southern California, Los Angeles, CA, USA; 3 Director, Movement Sciences Center, Institute for Rehabilitation Sciences and Engineering, Madonna Rehabilitation Hospital, Lincoln, NE; Adjunct Assistant Professor of Clinical Physical Therapy, Department of Biokinesiology and Physical Therapy, University of Southern California, Los Angeles, CA, USA; 4 Adjunct Associate Professor of Clinical Physical Therapy, Department of Biokinesiology and Physical Therapy, University of Southern California, Los Angeles, CA, USA; 5 Professor, Department of Orthopedic Surgery, Keck School of Medicine, University of Southern California, Los Angeles, CA, USA; 6 Long Beach Memorial Medical Center, Long Beach, CA, USA

## Abstract

**Background:**

Posterior tibialis tendon dysfunction (PTTD) is a common cause of foot pain and dysfunction in adults. Clinical observations strongly suggest that the condition is progressive. There are currently no controlled studies evaluating the effectiveness of exercise, orthoses, or orthoses and exercise on Stage I or IIA PTTD. Our study will explore the effectiveness of an eccentric versus concentric strengthening intervention to results obtained with the use of orthoses alone. Findings from this study will guide the development of more efficacious PTTD intervention programs and contribute to enhanced function and quality of life in persons with posterior tibialis tendon dysfunction.

**Methods/design:**

This paper presents the rationale and design for a randomized clinical trial evaluating the effectiveness of a treatment regime for the non-operative management of Stage I or IIA PTTD.

**Discussion:**

We have presented the rationale and design for an RCT evaluating the effectiveness of a treatment regimen for the non-operative management of Stage I or IIA PTTD. The results of this trial will be presented as soon as they are available.

## Background

Posterior tibialis tendon dysfunction (PTTD) is a common cause of foot pain and dysfunction in adults [1]. Clinical observations strongly suggest that the condition is progressive. Descriptively, the varying presentations of this condition are divided into four stages. Stage I is characterized by mild swelling and medial ankle pain but no deformity. Stage II is characterized by progressive flattening of the arch, with an abducted midfoot. In stage IIA, the foot is still flexible; however, the tendon is functionally impaired. In Stage IIB, the tendon is incompetent, or even ruptured. Stage III includes all of the signs of stage II; however, the hindfoot deformity becomes fixed. Myerson added Stage IV for patients who progressed to valgus tilt of the talus in the ankle mortise leading to lateral tibiotalar degeneration [2]. Despite its high prevalence [3], there are no intervention guidelines for Stage I or II PTTD, and surgical repair is the only definitive treatment for Stage III or IV. Factors associated with PTTD include the following: age-related degeneration, inflammatory arthritides [4,5], hypertension, diabetes mellitus, obesity, and less frequently acute traumatic rupture [2,5,6].

PTTD was previously thought to result from inflammatory processes; however, surgical exploration and tissue analysis has not confirmed the presence of inflammatory cells, such as macrophages. The histopathological findings are characterized by fibroblast hypercellularity, mucinous degeneration, and neovascularization [7]. It is thought that these changes result in disruption of the linear orientation of the collagen bundles, representing tendon degeneration and poor tissue response to healing [5]. Ultrasonography demonstrates increased tendon thickening, hypoechoic areas, irregular tendon structure with disrupted fibers, and synovial sheath effusion within a pathological posterior tibialis tendon [8,9]. The pathologies described above are characteristic of tendinosis, not tendinitis [7,10]. The consequences of the above findings have significant ramifications in the management of person's with PTTD. The emphasis of an intervention should therefore be on promotion of tissue remodeling and adaptation under optimal loading condition (rest, orthoses, specific exercise), and time (at least three months) [11]. This process requires effective patient education in regards to length of time to resolution, activity, consistent orthoses wear, exercise, and weight control [11]. Additionally, it is important that the patient understands that absence of pain does not reflect absence of degeneration.

Frequently, progressive exercise programs serve as a cornerstone for the non-surgical management of musculoskeletal pathologies. Recent studies demonstrated positive clinical results with eccentric calf muscle training in individuals with painful chronic Achilles tendinosis [12,13]. The participants were instructed in a concentric or eccentric training regimen performed on a daily basis for 12 weeks. Subjects in both groups were instructed to perform the exercises even if tendon pain or discomfort were experienced during exercise. Following completion of the 12-week program, 82% of the participants assigned to the eccentric group were satisfied with the results of the intervention and had resumed their previous activity level (before injury), compared to only 36% of those engaging in concentric training (p < 0.002) [13].

The current study in persons with tibialis posterior tendinopathy was motivated by the significant improvements documented for the eccentric training program for persons with Achilles tendinosis. The greater improvements demonstrated in response to an eccentric compared to a concentric strengthening program suggest that exercise interventions which incorporate an eccentric strengthening component may lead to more successful outcomes than concentric strengthening programs in the treatment of tendinosis. Our study will explore the effectiveness of an eccentric versus concentric strengthening intervention while wearing an orthoses to results obtained while using only an orthoses. There are no controlled studies evaluating the effectiveness of exercise, orthoses, or orthoses and exercise on PTTD. Findings from this research will be invaluable to PTTD intervention programs, as these data will provide critical information that will lead to more efficacious treatment interventions to enhance function and quality of life in persons with posterior tibialis tendon dysfunction.

## Methods

The University of Southern California Institutional Review Board granted approval for this study.

### Study population

Forty-five individuals with a current episode of medial ankle or foot pain who present to the Department of Orthopedics at the University of Southern California, Long Beach Memorial, or one of 17 physical therapy clinics participating in the Clinical Research Network of the Department of Biokinesiology and Physical Therapy at the University of Southern California will be recruited. All subjects will be screened for eligibility according to the inclusion and exclusion criteria by a research investigator. Only subjects presenting with Stage I or IIA PTTD will be enrolled in this study.

### Inclusion criteria

To be eligible for participation, subjects must meet one of the following criteria:

• pain at the medial ankle or foot reported for greater than three months duration

• tenderness localized to the tibialis posterior tendon

### Exclusion criteria

Participants will be excluded if they have one of the following conditions:

• fixed foot deformities

• previous foot surgery

• presence of any other concurrent foot pathology besides posterior tibial tendon dysfunction

• inability to walk without assistive device

• nervous system problems (e.g., stroke, dementia, seizures)

• cognitive dysfunction (e.g. TBI, CVA)

• uncontrolled cardiovascular disease

• evidence of cord compression

• uncontrolled hypertension

• infection

• severe respiratory disease

• pregnancy

• current or recent history of low back pain

• rheumatic joint disease

• peripheral vascular disease with sensory loss in the foot

• the subject identifies that she is pregnant

• any condition that the subject identifies which may limit participation in physical activity

### Outcome measures

The following outcome measures will be used in this randomized clinical trial:

#### Foot Functional Index (FFI)

The FFI is a self-reported measure of pain, function, and activity level associated with foot dysfunction consisting of 23 items divided into 3 sub-scales. The scores range from 0 to 10, with the higher scores indicating more disability. Both total and sub-scale scores are calculated [14]. The Foot Function Index (FFI) has been validated and determined to be a reliable instrument for patients with rheumatoid arthritis [14]. It is also recommended as a reliable measurement scale for use in other foot orthopedic interventions trials [15] and has been shown to be a reasonable tool for use with low functioning individuals with foot disorders [16].

#### Global Rating Scale (GRS)

The GRS is a self-reported measure of overall change in subject's condition resulting from a treatment intervention with respect to activity limitations, symptoms, emotions, and quality of life on a 15-point scale [17]. This scale has been shown to demonstrate minimal differences consistently across domains for both improvement and deterioration [18].

#### Physical Activity Scale (PAS)

The PAS is a self-reported evaluation of time spent performing daily activities such as sleep, rest, or physical activity (work, leisure time, and exercise activity) on a 24-hour scale. These data will be computed and represented in metabolic equivalents (METs). The PAS has been shown to be a valid self-reporting method for physical activity. It also has high correlation with the physical-activity diary, another self-reporting method of physical activity [19].

#### Short Form 36 Health Survey (SF-36)

The SF-36 was developed as a multi-purpose measure of health-related quality of life for use on general and specific populations without targeting a specific age, disease, or treatment group [20]. The SF-36 has proven useful in monitoring general and specific populations, comparing the burden of different diseases, differentiating the health benefits produced by different treatments, and in screening individual patient's health profile. The SF-36 was developed for multiple applications and populations so that the SF-36 scores from different samples could be compared [20].

#### 5-Minute walk test

The 5-minute walk test is a measure of function, endurance, and gait velocity. We developed and field-tested specific verbal instructions to provide consistency in testing. For the 5-Minute Walk Test the instructions read as follows: "This assessment involves walking as far as you comfortably can along this path for 5 minutes. Please let me know when you're ready and I'll give you the signals, "Ready" and "Go" to begin the timing. In order to keep the conditions identical for all the subjects, I will not be encouraging you during the walk, but I will let you know when each full minute has passed. You may stop and rest at any time during the assessment and continue walking when you're able. I'll record the total distance you cover over the 5-minute walk. Do you have any questions at this time?"

#### 50-Foot walk test

The Fifty-Foot Walk Test is a measure of gait velocity and function. A specific verbal instruction, similar to that presented in the 5-Minute Walk Test was developed and field-tested for this study.

#### Timed Up and Go (TUG)

The TUG is a test of basic functional mobility for elderly persons. A specific verbal instruction, similar to that presented in the 5-Minute Walk Test was developed and field-tested for this study.

#### Visual Analogue Scale for pain (VAS)

The VAS is a single dimension scale with endpoints marked as "no pain" and "worst pain possible." It is a reliable and valid measure of self-reported pain intensity [21]. The stability of a VAS score has been reported (r = 0.9) [22]. The VAS will be used to measure pain intensity after completion of the 5-Minute Walk Test, Fifty-Foot Walk, and Timed Up and Go.

### Evaluation

The research investigator performing both pre- and post-intervention evaluations will be standardized to the assessments and blinded to the subjects' group assignment.

### Timing of evaluation

#### Pre-intervention (PRE)

A standard orthopedic lower quadrant assessment documenting structural condition, mobility and strength, will be followed by the administration of the *Foot Functional Index, Physical Activity Scale, and SF-36 questionnaires*, and performance of a *5-Minute Walk Test, 50-Foot Walk Test*, as well as the *Timed Up and Go*. The *Visual Analogue Scale *for pain will be administered after each functional test.

#### Post-intervention (POST)

At the conclusion of the intervention, subjects will be re-evaluated following the same testing procedures and using the same tests/questionnaires as during the initial evaluation.

#### 6-Month follow up (6-MONTH FOLLOW UP)

Six months after the completion of the intervention, subjects will be asked to complete the *Foot Functional Index, the Physical Activity Scale, SF-36 questionnaire, and the Global Rating Scale*. A pre-addressed, stamped envelope will be included in the correspondence. Patients will also be contacted via phone to ensure a timely response.

### Intervention

The following interventions will be provided in four clinics located in the greater Los Angeles area:

#### Orthoses

At the time of initial evaluation, all subjects' feet will be assessed and measured for orthoses. The orthoses will be fabricated from a plaster mold obtained with the patient lying prone and the foot in a subtalar joint neutral position, using procedures described by Biomechanical Service (1050 Central Ave. Suite D, Long Beach, CA 92832, 1-800-942-2272). The same company will fabricate the orthoses. The final design of the orthoses will be posted according to the amount of rearfoot pronation when comparing relaxed subtalar joint neutral and relaxed standing, (posting of 50–75% of the difference between these two positions). Additional, in the presence of a bony forefoot deformity, the forefoot will also be posted at 50% of the deformity. The subjects will be asked to comply with the protocol by wearing the orthoses 90% of their waking hours in athletic shoes.

#### Stretching

**Figure 1:**
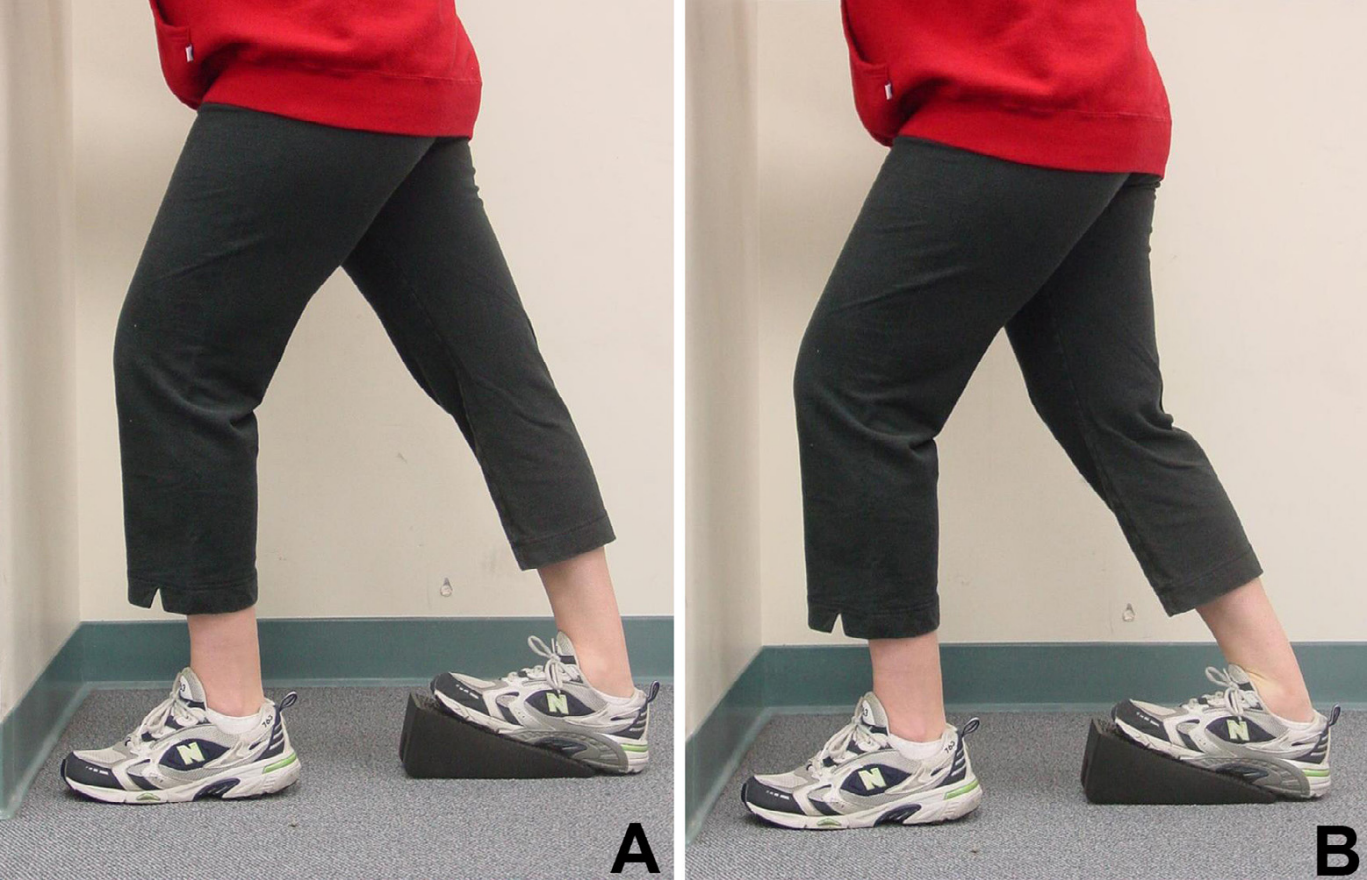
A) Standing gastrocnemius stretch. B) Standing soleus stretch.

All participants will perform calf stretches (Figure 1). Each subject will be issued a "Slant by OPTP" (OPTP, PO Box Minneapolis, MN 55447-0009) to be used for calf stretching. The slant is a lightweight, portable foam wedge. The subjects will be instructed to place the slant facing away from a wall, within a foot length distance. They will then place the shod foot of the "to be stretched leg" on the slant with the toes pointing up. The subject will be instructed to lean forward until he/she perceives a strong but tolerable stretch in the calf muscles. This maneuver will be repeated 3 times with the knee extended to target the gastrocnemius muscle and 3 times with the knee slightly flexed to more selectively isolate the soleus muscle. This position will be held for 30 seconds. The lumbar spine will be placed in a "neutral" position to reduce the potential risk of strain to the low back region. Additional fine points of the technique, specific to the needs of individual subjects, will be taught to the patient by the intervention therapist. The subjects will receive a pictorial and written description of the stretching technique.

#### Progressive resistive exercise

**Figure 2:**
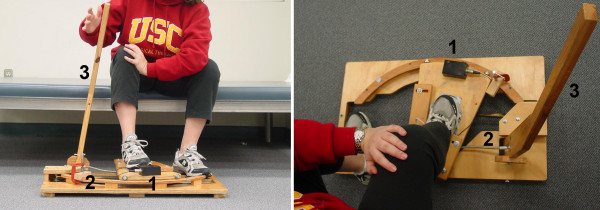
Exercise unit for isolated tibialis posterior exercise. (1) LED displaying static plantar flexion from pressure sensors under forefoot; (2) Constant force extension spring for dynamic adduction; (3) Lever to allow passive adduction or abduction of the foot.

Previous work has indicated that the tibialis posterior is preferentially recruited during a resisted foot adduction exercise in persons with pes planus [23] and that this muscle is selectively activated when flat-footed subjects perform the exercise while wearing arch supporting orthoses and shoes [24]. Therefore, the exercises will consist of isolated loading (plantar flexion and adduction) of the posterior tibialis musculotendinous unit and will be performed with the subjects wearing both orthoses and shoes. The exercises will be performed using a specialized exercise unit, which can be manipulated to progressively load the tendon either concentrically or eccentrically depending on group assignment (Figure 2). Subjects will begin resistive exercise when custom orthoses are delivered (1–2 weeks after evaluation).

### Intervention group assignment

Subjects will be randomly assigned to one of the three groups: 1) orthoses wear and calf stretching; 2) orthoses wear, calf stretching, and a progressively challenging concentric exercise program; 3) orthoses wear, calf stretching, and a progressively challenging eccentric exercise program.

#### Orthoses (ORTH)

Subjects in the orthoses group will wear custom foot orthoses in athletic shoes for 90% of waking hours. They will be instructed in performing calf stretches as described earlier. A series of six, 30-second stretches on the involved leg will be performed twice daily. Subjects will begin to perform calf stretches on the day of initial evaluation.

#### Orthoses + concentric exercise program (CONC)

Subjects in the concentric exercise group will wear custom foot orthoses and perform calf stretches as described for the orthoses group. They will also be instructed in the performance of the concentric exercise program (consisting of plantar flexion and adduction using a specialized exercise unit). The exercise will be performed slowly (5 seconds thought the range of motion). A series of 3 sets of 15 repetitions will be performed twice daily on the involved side. Between sets, rest periods will be 1–2 minutes. The resistance, provided by a constant force extension spring, will be set at 2 pounds for the first week and progressed to tolerance and ability throughout the 10-week concentric intervention program.

#### Orthoses + eccentric exercise program (ECC)

Subjects in the eccentric exercise group will wear custom foot orthoses and perform calf stretches as described for the orthoses group. They will also be instructed in the performance the eccentric exercise regimen using a specialized exercise unit, which requires plantar flexion and resistance to adduction. The exercise will be performed slowly (5 seconds throughout the range of motion). A series of 3 sets of 15 repetitions will be performed twice daily on the involved side. Between sets, rest periods will be 1–2 minutes. The resistance, provided by a constant force extension spring, will be set at 2 pounds for the first week and progressed to tolerance and ability throughout the 10-week eccentric intervention program.

Subjects will have an Exercise Record Chart where they will record the number of performed stretches and exercises as well as reflections on their performance (ease; if discomfort, then location and intensity). Subjects will meet with an intervention therapist once per week for 10 weeks so that the quality of stretches and motion during the exercise can be assessed and resistance can be added as tolerated or decreased as needed.

### Data analysis

A 3 × 3 ANOVA with repeated measures will identify differences in the FFI across groups (ORTH, CONC, ECC) and between testing sessions (PRE, POST, 6-MONTH FOLLOW UP). All analyses will be performed using SPSS statistical software (SPSS Inc., Chicago, IL). All significance levels will be set at p < 0.05.

### Sample size

The selection of 45 subjects for this study (15 subjects per group), is based on clinical observations, attrition rate, and power analysis. Clinical observations have indicated that the inter-subject variability with respect to pain, disability and function is moderate to high. Therefore, all power calculations have taken this increased variability into consideration. A 20% subject attrition rate is anticipated. The possible attrition rate is attributed to length of the rehabilitative program, elimination of symptoms, or increase of symptoms, need for surgery, and prior attrition rate of existing randomized control trials. The *Foot Functional Index *will be used as the primary outcome of this study. The remaining functional, health status, and pain variables will be used as secondary outcome variables. Power calculations were based on an alpha level of 0.05 and beta of 0.8 with respect to the FFI pain score.

## Discussion

We have presented the rationale and design for an RCT evaluating the effectiveness of a treatment regimen for the non-operative management of Stage I or IIA PTTD. The results of this trial will be presented as soon as they are available.

## Competing interests

The author(s) declare that they have no competing interests.

## Authors' contributions

JMB, KK, SFR, and SMR were responsible for the design of the study. ABP is the study coordinator and prepared the current manuscript. RWS and DBT contributed to the conceptual framework of the study. All authors read and approved the final manuscript.

## Pre-publication history

The pre-publication history for this paper can be accessed here:
